# Spatial patterns of prematurity and its determinants in the metropolitan region of São Paulo, Brazil, 2010-2019

**DOI:** 10.1590/1980-549720240008

**Published:** 2024-02-26

**Authors:** Elias Carlos Aguirre Rodríguez, Elen Yanina Aguirre Rodríguez, Fernando Augusto Silva Marins, Aneirson Francisco da Silva, Luiz Fernando Costa Nascimento

**Affiliations:** I Universidade Estadual Paulista “Júlio de Mesquita Filho”, Postgraduate Program in Engineering - Guaratinguetá (SP), Brazil.; II Universidade de Taubaté, Postgraduate Program in Environmental Sciences - Taubaté (SP), Brazil.

**Keywords:** Premature birth, Prematurity, Spatial analysis, Geographic information systems, Geographic mapping, Nascimento prematuro, Prematuridade, Análise espacial, Sistemas de informação geográfica, Mapeamento geográfico

## Abstract

**Objective:**

To analyze spatial distribution of preterm births and their association with maternal, social, and health services indicators in the metropolitan region of São Paulo, Brazil, 2010-2019.

**Methods:**

Ecological study using data on preterm newborns from 39 municipalities in the metropolitan region of São Paulo. Univariate global Moran’s index (Im) was used to evaluate spatial association of prematurity, and univariate local Moran’s index by using the cluster map (LISA) to identify spatial patterns and clusters. Bivariate global Moran’s index was also used to analyze spatial autocorrelation with maternal, social, and health services indicators.

**Results:**

A total of 3,103,898 live births were registered in period 2010-2019, of which 331,174 (10.7%) were preterm. The global Moran’s index showed spatial independence (Im=0.05; p-value=0.233) of the proportion of preterm births between municipalities. However, in the local spatial analysis it was possible to identify a statistically significant spatial cluster between the municipalities of Biritiba Mirim, Guararema and Salesópolis, with high proportions of preterm births. In the bivariate analysis, a significant positive spatial association was identified with proportions of mothers under 20 years old (Im=0.17; p-value=0.024) and mothers with low schooling (Im=0.17; p-value=0.020), and a significant negative spatial association with HDI (Im=-0.14; p-value=0.039).

**Conclusions:**

The local spatial approach identified a spatial cluster located in the far east of the metropolitan region of São Paulo, where actions by health managers are needed to minimize occurrence of preterm births.

## RESUMO

**Objetivo:**

Analisar a distribuição espacial dos nascimentos prematuros e sua associação com indicadores maternos, sociais e de serviços de saúde na região metropolitana de São Paulo, Brasil, 2010-2019.

**Métodos:**

Estudo ecológico utilizando dados sobre recém-nascidos pré-termo dos 39 municípios da região metropolitana de São Paulo. Utilizou-se o índice de Moran (Im) global univariado para avaliar a associação espacial da prematuridade, e o índice de Moran local univariado por meio do mapa de clusters (LISA) para a identificação de padrões e aglomerados espaciais. Também foi utilizado o índice de Moran global bivariado para analisar a autocorrelação espacial com os indicadores maternos, sociais e de serviços de saúde.

**Resultados:**

Foram registrados 3.103.898 nascidos vivos no período 2010-2019, dos quais 331.174 (10,7%) foram prematuros. O índice de Moran global mostrou independência espacial (Im=0,05; p-valor=0,233) da proporção dos nascimentos prematuros entre municípios. No entanto, na análise espacial local foi possível identificar aglomerado espacial estatisticamente significativo entre os municípios de Biritiba Mirim, Guararema e Salesópolis, com proporções altas de nascimentos pré-termo. Na análise bivariada, identificou-se associação espacial significativa positiva com proporções de mães menores de 20 anos (Im=0,17; p-valor=0,024) e mães com baixa escolaridade (Im=0,17; p-valor=0,020), e associação espacial significativa negativa com IDH (Im=-0,14; p-valor=0,039).

**Conclusão:**

A abordagem espacial local identificou agrupamento espacial situado no extremo leste da região metropolitana de São Paulo, onde ações dos gestores de saúde são necessárias para minimizar a ocorrência de partos prematuros.

## INTRODUCTION

Premature birth, also called preterm, occurs before the 37^th^ week of gestation and, according to the World Health Organization (WHO), more than 10% of newborns present this condition in 2020^1^. Furthermore, prematurity is associated with a greater risk of physical and neuropsychomotor complications in newborns^2^. It is considered one of the main causes of mortality, especially in the neonatal period (during the first 27 days of life), causing around 1.1 million newborn deaths annually^1^ ^,^ ^3^.

In this scenario, through the Millennium Development Goals (MDG), WHO member states committed to reducing under-five mortality by two thirds by 2015, however, the results achieved were not sufficient to achieve this goal^4^. With the Sustainable Development Goals (SDG), in 2016, new objectives and targets came into force, establishing that, by 2030, there will be a reduction of under-five mortality by at least 25 per 1,000 live births and neonatal mortality in at least 12 per 1,000 live births^5^.

Therefore, reducing prematurity and its determinants is one of the keys to reducing the number of deaths, especially in the neonatal period, given the relationship between mortality and prematurity and its consequences, such as low birth weight^6^ and other complications^7^.

Noteworthily, Brazil occupies the 10^th^ position among the countries with the highest number of premature births, the 2^nd^ position in the American continent, after the United States, and the 1^st^ position in Latin America^1^. Additionally, according to the National Early Childhood Network, premature birth in Brazil represents a cost of approximately R$8 billion per year^8^. Furthermore, after birth, newborns remain in intensive care for an average of 51 days, which costs more than R$15 billion annually, making it paramount to improve hospital care for mothers and premature newborns^9^ ^,^ ^10^.

There are multiple factors involved in prematurity, including determinants that are the responsibility of the health sector, which address biological aspects, as well as those determinants related to social, political, economic or environmental aspects, which are considered a responsibility of the State^11^.

Tools such as geoprocessing are available in the literature, which are used to identify, locate, monitor, and follow-up populations, enabling the development of studies to control and monitor diseases in a given area^12^. With geoprocessing methods, such as spatial analysis, it is possible to identify risk zones and any associated factors, making it a great ally of public health^13^.

Among the studies where geoprocessing was used, the analyses on the profile of births^14^, morbidity^7^ ^,^ ^15^, and neonatal, post-neonatal (28 to 364 days of life) or infant mortality (under 1 year of life)^6^ stand out^16^ ^,^ ^17^. Specifically, studies were found on the spatial distribution of premature births in Brazil^7^ ^,^ ^18^ ^,^ ^19^ and in other countries^20^ ^,^ ^21^ ^,^ ^22^ ^,^ ^23^, whose risk areas were identified and spatial associations with some factors were studied, such as those related to health^7^, industry^21^, agriculture^22^, the environment^23^ or some socioeconomic conditions^19^.

In this context, this research was developed with the objective of analyzing the distribution and spatial autocorrelation of premature live births in the Metropolitan Region of São Paulo (MRSP), between 2010 and 2019. Furthermore, the degree of spatial association between prematurity and different determinants such as maternal, social, and service indicators was explored, with the aim of identifying possible spatial groupings of municipalities that require some intervention to minimize the risk of a premature newborns.

## METHODS

The study carried out was ecological, with data from secondary sources on preterm live births in MRSP between 2010 and 2019.

**Figure 1.**
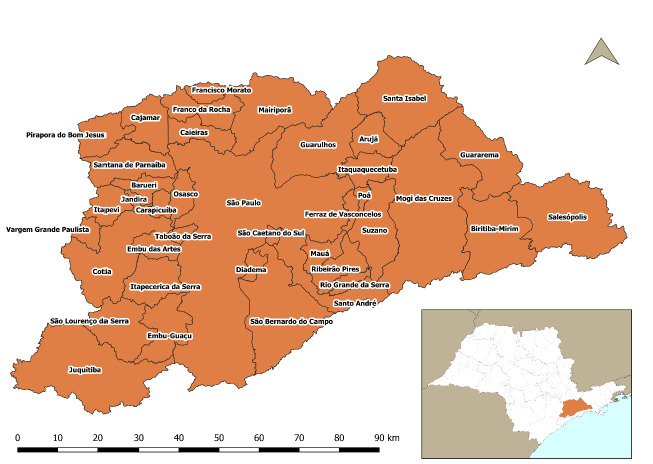
Map of the political-administrative division of municipalities in the metropolitan region of São Paulo.

MRSP is made up of 39 municipalities (Figure 1) and has an extension of 7,946.98 km^2^, representing a little more than 3% of the state of São Paulo (249,219.94 km^2^), with more than 21 million inhabitants^24^.

Figure 1 represents the digital map of the analyzed area, obtained from the Brazilian Institute of Geography and Statistics (*Instituto Brasileiro de Geografia e Estatística* - IBGE)^25^ portal, showing the municipalities belonging to MRSP.

The database containing records of premature live births (<37 weeks of gestation), based on the mother’s municipality of residence, was obtained from the Live Birth Information System (*Sistema de Informação de Nascidos Vivos* - SINASC), through the Information Technology Department of the Unified System of Health (*Departamento de Informática do Sistema Único de Saúde* - DATASUS) and made available by the information system of the Ministry of Health^26^.

The proportions of premature births were calculated by dividing the number of preterm live births with the total number of live births in each municipality in the period 2010-2019, and expressed per 100 live births.

For the analysis, variables related to different determinants were also calculated, classified as maternal, social, and health service indicators. Note that, according to the reviewed literature, these variables have already been analyzed in regard to spatial patterns of mortality in full-term and low birth weight newborns^6^, but not based on the spatial approach proposed here for prematurity. These determinants are easy to obtain and are available on official Brazilian websites such as DATASUS^26^.

 Maternal indicators were: proportion of mothers aged under 20 years old (adolescents), proportion of mothers aged >34 years old (advanced age), and proportion of mothers with up to 7 years of study (low education), obtained from SINASC-DATASUS^26^ and calculated in relation to the total number of premature live births in each municipality in the 2010-2019 period.

Social indicators were obtained from the repository of the Institute for Applied Economic Research (*Instituto de Pesquisa Econômica Aplicada* - IPEA), namely: the Social Vulnerability Index (SVI), provided through the Social Vulnerability Atlas in Brazilian municipalities and metropolitan regions^27^; the Human Development Index (HDI) and the GINI Index, made available by the Human Development Atlas in Brazil^28^, based on data acquired in the 2010 census.

Indicators related to health services were: rate of gynecologists-obstetricians, rate of doctors from the Family Health Strategy, and rate of obstetric beds, which were acquired from the National Registry of Health Establishments (*Cadastro Nacional de Estabelecimentos de Saúde* - CNES) in DATASUS^26^ and calculated as the average of monthly data per municipality in the 2010-2019 period, considering 100,000 inhabitants.

The proportions of premature births by municipality were represented through thematic maps, using quartile categorization.

To analyze spatial autocorrelation, the univariate global Moran index (I_m_) was used to verify the degree of spatial association of the proportions of premature births in the municipalities of MRSP, at levels of statistical significance with p-value <0.05.

The univariate global Moran index (I_m_) can have values between [-1, +1], indicating a positive spatial autocorrelation (0<I_m_≤1), where municipalities with high values in the proportions of premature births tend to cluster together, and similarly for municipalities with low values^23^. A negative spatial autocorrelation (-1≤I_m_<0) is observed when municipalities with high proportions of premature births tend to be very close to municipalities with low values and vice versa, as well as the absence of spatial autocorrelation can also be observed (spatial independence, I_m_=0)^6^ ^,^ ^29^.

Additionally, the Local Indicator of Spatial Association (LISA) cluster map was used to identify local patterns of spatial autocorrelation, allowing the location of spatial groupings by type of association (High-High, High-Low, Low-High, and Low-Low), according to the statistical significance test of the univariate local Moran index^30^.

The bivariate global Moran index (I_m_) was used to identify the presence of spatial autocorrelation between the proportion of premature births and each of the different indicators studied in the municipalities of MRSP. Likewise, its values vary between [-1, +1], indicating the existence of an inverse or direct spatial autocorrelation between the values of two analyzed variables^6^ ^,^ ^29^.

Public access software such as QGIS v.3.26.0 (https://qgis.org/pt_BR/site/) were also used to generate digital maps of the studied area, and GeoDA v.1.20.0 (https://geodacenter.github.io/), for data processing through spatial autocorrelation analysis.

The study was carried out with secondary data sources made available by public domains DATASUS, IBGE, and IPEA, therefore it was not necessary to submit the project to a Research Ethics Committee, based on Resolution of the National Health Council No. 510/16, of April 7^th^, 2016.

## RESULTS

**Figure 2.**
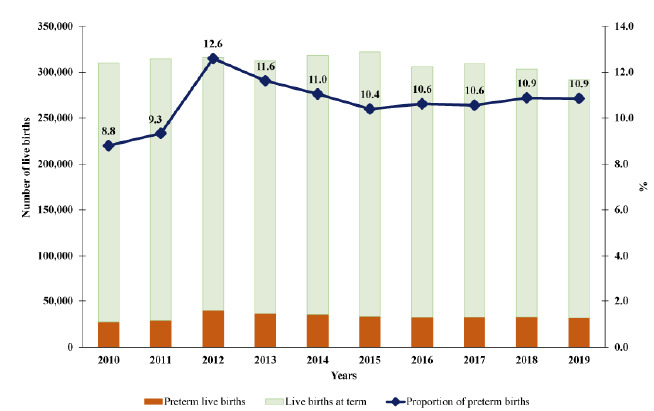
Annual distribution of live births and proportion of preterm births in the metropolitan region of São Paulo, 2010-2019.

Between 2010 and 2019, 3,103,898 live births were recorded in the study area, of which 331,174 (10.7%) were preterm births. Thus, Figure 2 shows that, in 2010, the proportion of premature births was 8.8%, with an increase of almost 4 percentage points (pp) in 2012, reaching 12.6%. A slight decrease in the proportion of premature births could be observed in the following years, reaching 10.9% of premature births in 2019, which represents an increase of approximately 2pp in this period.

**Figure 3.**
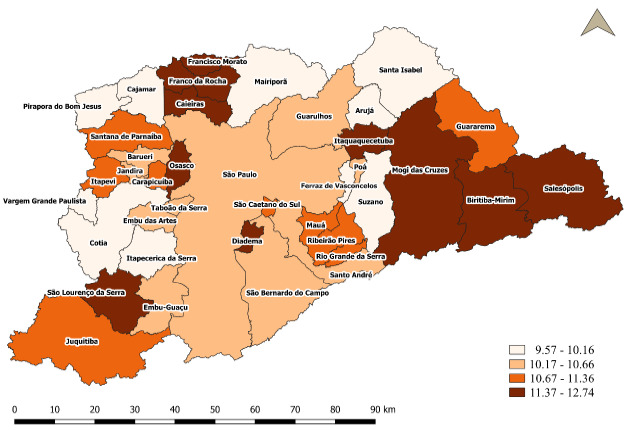
Spatial distribution of the proportion of preterm births in the municipalities of the metropolitan region of São Paulo, 2010-2019.

Analysis of the spatial distribution for the 2010-2019 period in MRSP, considering quartiles, showed that the proportion of preterm births varied from 9.57 to 12.74 premature births per 100 live births, where 51.3% (20) of the municipalities had proportions lower than and equal to 10.66 premature babies per 100 live births and 48.7% (19) of the remaining municipalities had proportions above 10.66 premature babies per 100 live births (Figure 3).

**Figure 4.**
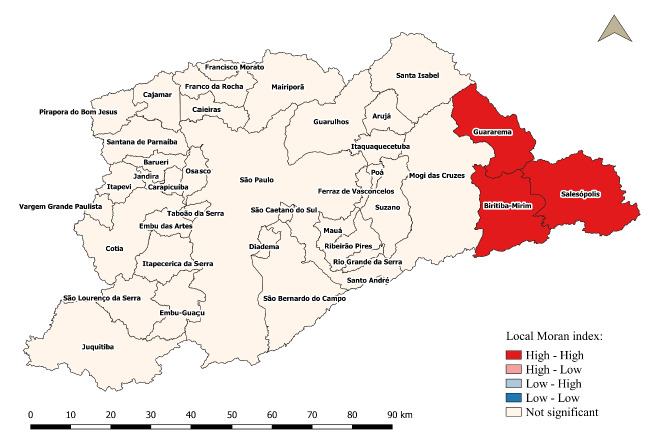
Cluster map (LISA) of the proportion of preterm births in the municipalities of the metropolitan region of São Paulo, 2010-2019.

Spatial autocorrelation, determined by the global Moran index for the proportion of premature births, was I_m_=0.05 with p-value=0.233, indicating that there is spatial independence between municipalities (absence of spatial autocorrelation). However, according to the statistical significance test of the univariate local Moran index, the cluster map (LISA) shows the existence of a spatial cluster located in the extreme east of the region. Specifically, the cluster formed by the municipalities of Biritiba Mirim, Guararema and Salesópolis, which presented high proportions of premature births (High-High), while the other municipalities did not show a significant association (Figure 4).

Based on bivariate Moran analysis (Table 1), it was possible to identify the degree of association and statistical significance between the proportion of premature births and maternal, social, and health service indicators.

**Table 1.** Bivariate Moran Index (I_m_) between the proportion of preterm births and maternal, social, and health service indicators.

Characteristics	I_m_	p-value
Maternal determinants		
Mothers aged <20 years old (%)	0.17	0.024*
Mothers aged >34 years old(%)	-0.12	0.062
Mothers with ≤7 years of education (%)	0.17	0.020*
Social indicators		
Social Vulnerability Index (SVI)	0.02	0.371
Human Development Index (HDI)	-0.14	0.039*
GINI Index	-0.03	0.372
Health service indicators		
Gynecologist-Obstetrician doctors per 100,000 inhabitants	0.01	0.453
Family Health Strategy doctors per 100,000 inhabitants	-0.09	0.103
Obstetric beds per 100,000 inhabitants	0.09	0.108

*Significant (p-value<0.05).

The proportion of premature births showed a positive and significant spatial association with maternal indicators, such as the proportion of mothers aged under 20 years old (I_m_=0.17; p-value=0.024) and the proportion of mothers with up to 7 years of schooling (I_m_=0.17; p-value=0.020). A negative and significant spatial association was also identified with the social indicator HDI (I_m_=-0.14; p-value=0.039).

No significant spatial associations were found between the proportion of premature births and the proportion of mothers aged over 34 years old, SVI, GINI Index and any of the health service indicators (rate of gynecologists-obstetricians, rate of doctors from the Family Health Strategy, and obstetric bed rate).

## DISCUSSION

The study, through spatial analysis, found that there is global spatial independence for the proportion of premature births among the municipalities in MRSP between 2010 and 2019. However, local assessment identified the presence of a unique grouping of the municipalities of Biritiba Mirim, Guararema, and Salesópolis, which had high values in the proportions of preterm births, basically located in the eastern end of MRSP. Furthermore, there was a significant positive spatial association with the proportion of mothers under 20 years of age and mothers with low education, and a significant negative spatial association with HDI.

In 2010, around 15 million newborns worldwide were premature, which represented 11.1% of live births, with a variation of around 5% in European countries and up to 18% in some African countries^1^ ^,^ ^31^. It can therefore be seen that, in low-income countries, the proportion of premature newborn deaths is greater than 90% in the first days of life, while in high-income countries it is less than 10%^1^.

The study of the trend of prematurity between 2000 and 2014 showed an increase in the proportions of premature births, increasing from 9.8 to 10.6% worldwide^32^. However, in Brazil, the proportions of preterm births showed a decreasing trend from 2012 to 2019, ranging from 10.9 to approximately 10%^33^.

Also, in MRSP, it was observed that, in the 2000-2010 decade, the proportions of premature births showed an increasing growth with values that ranged between 7.5 and 8.8%, with a mean of 8.1%^19^. This research showed that from 2010 to 2019, the average proportion of preterm births was 10.7%, representing an increase of approximately 2.6 pp (compared to the 2000-2010 period). It was also found that the highest proportion (12.6%) was obtained in 2012 and that subsequently there was a slight decrease, which reached 10.9% of premature births in 2019.

Likewise, compared to the North and Northeast regions of Brazil, the proportions of premature births were 11 and 10.5%, respectively, with only a minimal difference in relation to the studied MRSP^26^.

As spatial analysis has been a good way to carry out epidemiological studies by mapping various problems such as prematurity, Bloch^20^ used a spatial approach through the census in Philadelphia to study the geographic pattern of premature births to black mothers born abroad and in the United States, and systematic variation across communities was observed in race, poverty, and crime. Another study carried out by Castelló et al.^21^, in Spain, also showed spatial variations in the risk of prematurity and low birth weight, highlighting some areas with high risk that could be related to industry or agriculture, in addition to other factors, such as vulnerability unequal social status.

In China, Li et al.^22^ found global spatial dependence of the relative risks of prematurity (I_m_=0.40; p-value<0.050), identifying spatial groupings of areas with a high risk of premature births that coincided with areas of high consumption of fertilizers. Likewise, Genin et al.^23^ observed spatial independence in the distribution of the incidence of premature births (I_m_=0.09; p-value=0.001) in France, in addition to identifying a significant positive association with the environmental score related to air pollution.

Marinonio et al.^7^, in the state of São Paulo (Brazil), identified spatial dependence in the rates of preterm live births (I_m_=0.78; p-value=0.001) and in the rates of neonatal deaths associated with respiratory distress syndrome (I_m_=0.73; p-value=0.001), in addition to detecting spatial clusters with municipalities that had high rates of neonatal mortality associated with respiratory distress syndrome and that were close to municipalities with low rates of preterm live births, and vice versa. Furthermore, Paulucci et al.^18^ examined premature births in the city of Taubaté, identifying a positive spatial autocorrelation (I_m_=0.07; p-value=0.02) and the formation of clusters of some census tracts that require intervention.

In the comparative study of the relative risk of preterm births and its association with socioeconomic variables between MRSP in Brazil and the Metropolitan Area of Lisbon in Portugal (MAL) for the 2000-2010 period, carried out by Miranda et al.^19^, spatial dependence of the relative risk of preterm births was identified in the two geographic areas, a result very different from that found in this study, which was spatial independence. In MRSP, from 2000 to 2010, the existence of a negative spatial autocorrelation was verified between relative risk and unemployment rate (I_m_=-0.23; p-value=0.018), and in MAL, a positive spatial autocorrelation between relative risk and illiteracy rate (I_m_=0.44; p-value=0.007), unemployment rate (I_m_=0.26; p-value=0.033), and sociomaterial deprivation index (I_m_=0.35; p-value=0.014)^19^.

Thus, premature birth is the result of a complex relationship between multiple factors, including biological, maternal, social, economic, and health service determinants^11^ ^,^ ^34^. In this study, the spatial association of premature births with some maternal and social determinants, such as the age and level of education of mothers, in addition to the HDI inequity indicator, was verified.

The analysis showed that maternal age below 20 years old showed a positive spatial association with premature births, which means that teenage mothers have a greater risk of premature birth; on the other hand, maternal age over 34 years old did not show spatial autocorrelation. Likewise, the proportion of mothers with low education (up to 7 years) showed a positive spatial association with prematurity, indicating an association between low education and the occurrence of preterm newborns. These results were also found by Alberton et al.^35^ who evaluated prematurity rates in Brazil and the relationship with age thresholds and mothers’ low education.

Maternal age is a factor with a direct effect on prematurity, as teenage or older mothers can experience complications during pregnancy and childbirth, even causing other complications in the fetus. It can also be considered that mothers with inadequate education are twice as likely to have a premature birth, being associated and determined by living or socioeconomic conditions^36^.

Of the social indicators, only HDI showed a negative spatial association with premature births, which may be derived from social inequality and access to health services. Thus, if socioeconomic conditions were adequate, in order to increase HDI, this could generate a decrease in preterm newborns.

In this new scenario, with improvements in health, education, and income parameters, municipalities would have more adequate development and the risk of premature births would be reduced. It is noteworthy that SVI, the GINI Index, and health service indicators were not associated with prematurity.

Therefore, as I_m_ values are close to zero, it is important to mention that the associations found only partially explain prematurity, with the possibility of other factors, such as biological or socioeconomic, related to prematurity.

Even so, the research results corroborated what was described in some studies that suggest the implementation of public policies and specific strategies for the prevention and treatment of prematurity, such as offering interventions to improve the needs of women of reproductive age, including sexual education programs, in addition to improving the quality of prenatal care, as well as during and after childbirth^7^ ^,^ ^11^ ^,^ ^18^ ^,^ ^20^ ^,^ ^34^ ^,^ ^36^.

Based on the information resulting from this research, with the identification of the cluster of municipalities with high rates of prematurity, it may be that these municipalities have limited access to family planning programs or even a lack of professionals or inputs.

Thus, there is a need to formulate social programs, such as the provision of subsidies and educational campaigns, specifically considering the implementation of health policies, with intervention strategies for the prevention, control, and reduction of premature births in the highest risk municipalities, thus achieving a reduction in neonatal mortality rates, aiming to achieve the SDG targets by 2030.

Use of data from secondary sources, essentially made available by DATASUS, only for municipalities (not available for smaller areas, such as districts), whose quality can be affected by incomplete or misregistered information for some of the variables collected, referring to health service indicators obtained from the CNES stands out as a limitation of this work. In any case, a single consolidated database was used for the 2010-2019 period.

Even in view of the limitation previously mentioned and with spatial independence (absence of global spatial autocorrelation), the local spatial approach allowed identifying the cluster formed by the municipalities of Biritiba Mirim, Guararema, and Salesópolis, located in the extreme east of the metropolitan region of São Paulo, which had the highest proportions of premature births. This provides important information to municipal and regional health managers for timely intervention in areas with the highest risk of prematurity.
